# Genomic Rearrangements of *PTEN* in Prostate Cancer

**DOI:** 10.3389/fonc.2013.00240

**Published:** 2013-09-17

**Authors:** Sopheap Phin, Mathew W. Moore, Philip D. Cotter

**Affiliations:** ^1^Genetics, ResearchDx, Irvine, CA, USA; ^2^Department of Pediatrics, University of California San Francisco, San Francisco, CA, USA

**Keywords:** prostate cancer, PTEN, genomic rearrangements, fluorescence *in situ* hybridization

## Abstract

The phosphatase and tensin homolog gene (*PTEN*) on chromosome 10q23.3 is a negative regulator of the PIK3/Akt survival pathway and is the most frequently deleted tumor suppressor gene in prostate cancer. Monoallelic loss of *PTEN* is present in up to 60% of localized prostate cancers and complete loss of *PTEN* in prostate cancer is linked to metastasis and androgen-independent progression. Studies on the genomic status of *PTEN* in prostate cancer initially used a two-color fluorescence *in situ* hybridization (FISH) assay for *PTEN* copy number detection in formalin fixed paraffin embedded tissue preparations. More recently, a four-color FISH assay containing two additional control probes flanking the *PTEN* locus with a lower false-positive rate was reported. Combined with the detection of other critical genomic biomarkers for prostate cancer such as *ERG*, *androgen receptor*, and *MYC*, the evaluation of *PTEN* genomic status has proven to be invaluable for patient stratification and management. Although less frequent than allelic deletions, point mutations in the gene and epigenetic silencing are also known to contribute to loss of PTEN function, and ultimately to prostate cancer initiation. Overall, it is clear that PTEN is a powerful biomarker for prostate cancer. Used as a companion diagnostic for emerging therapeutic drugs, FISH analysis of *PTEN* is promisingly moving human prostate cancer closer to more effective cancer management and therapies.

## Introduction

Prostate cancer is one of the leading causes of cancer mortality in men in the Western world. In the United States, it is the most commonly diagnosed cancer in men and second only to lung cancer in the number of male cancer deaths (1). Prostate cancers display a variable range of clinical behaviors, from slow-growing tumors of little clinical significance to aggressively metastatic and lethal diseases. Current prognostic tools, such as pre-operative prostate specific antigen (PSA) levels, histological Gleason grading, clinical tumor, node, and metastasis (TNM) staging are used to place men in low-, intermediate-, and high-risk prostate cancer risk groupings. However, these prognostic tools often fail to accurately stratify individual patients at early stages of the disease. Although <5% of patients exhibit advanced disease, up to 40% of patients will eventually develop metastatic disease despite local therapy (2). Localized cancers are usually treated with radical prostatectomy or radiation. For more advanced cancers that have either recurred or metastasized, the gold standard treatment is androgen ablation therapy. Androgens play a central role in the normal development and growth of the prostate gland as well as the abnormal growth of prostate cancer. Androgen ablation by either surgical castration, or luteinizing hormone-releasing hormone (LHRH) analog treatments strongly inhibit the growth of localized advanced cancer by eliminating circulating testosterone (3, 4). Although very efficient at reducing cancer growth, this treatment eventually selects for cells that are no longer responsive to such therapy, resulting in a recurring lethal cancer within 18–24 months. This recurrent cancer is often referred to as Castration Resistant Prostate Cancer (CRPC) (5).

Given the broad spectrum of clinical and molecular behaviors, the wide range of clinical outcomes and their associated treatments, it is clear that prostate cancer is a highly heterogeneous disease that presents great complexities in determining risk stratification and appropriate treatment strategies. The main challenge for physicians remains to distinguish indolent from clinically significant tumors. With the goal of improving clinical management of the disease, current efforts are focusing on identifying the genes and understanding the pathways involved in mediating disease progression and treatment resistance. A further short term goal of genetic testing of tumor samples is the identification of appropriate companion diagnostics, allowing stratification of patients for treatment and monitoring of treatment.

## Genetic Alterations in the PI3K/Akt/mTOR Signaling Pathway in Prostate Tumorigenesis

**Figure 1:**
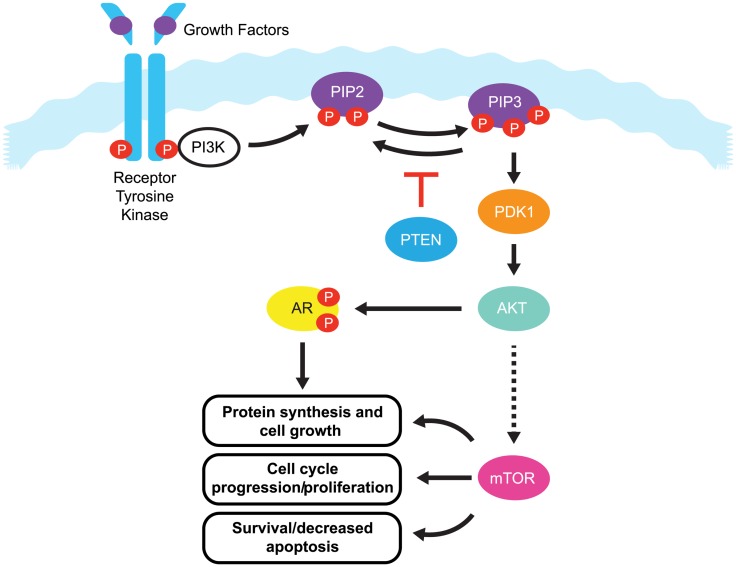
**The PI3K/PTEN/Akt pathway**. Binding of growth factors to the receptor tyrosine kinase activates the receptor complex, which in turn recruits and activates PI3K. Activated PI3K converts PIP2 to PIP3, which subsequently mediates the phosphorylation of Akt through PDK1. Phosphorylated Akt is active on a wide range of substrates, but one of its most important targets is mTOR, which is involved in cell growth, proliferation, and survival. Activated Akt also interacts with androgen receptor (AR) in an androgen-independent manner, leading to over-activation of the AR signaling pathway in castration resistant prostate cancer. PTEN is a tumor suppressor that negatively regulates the pathway by removing the 3-phosphate from PIP3, converting it back to PIP2. Loss of PTEN leads to over-activation of Akt which, in turn, is associated with uncontrolled cell proliferation, decreased apoptosis, and enhanced tumor angiogenesis.

One pathway with a prominent role in prostate cancer is the phosphatidylinositide 3-kinases (PI3K) signaling pathway. Current estimates suggest that this signaling pathway is up-regulated in 30–50% of prostate cancers (6–8). PI3K signaling is initiated by the activation of a number of receptor tyrosine kinases, including platelet-derived growth factor receptor (PDGFR), insulin-like growth factor receptor (IGFR), and epidermal growth factor receptor (EGFR) (Figure 1).

Once activated, these receptors phosphorylate PI3K at the cell membrane. Phosphorylated PI3K in turn phosphorylates phosphatidylinositol-4,5-diphosphate (PIP2), leading to the accumulation of phosphatidylinositol-3,4,5-triphosphate (PIP3). PIP3 recruits Akt (also known as protein kinase B) and phosphoinositide dependent protein kinase 1 (PDK1) to the cell membrane, where Akt is phosphorylated by PDK1. Phosphorylated Akt activity extends over a wide range of substrates, but most importantly activates the mammalian target of rapamycin (mTOR), which play a significant role in tumorigenesis (9). mTOR is a serine/threonine kinase that plays critical roles in the regulation of cell growth, survival, and division. Interaction between Akt and androgen receptor (AR) can lead to AR activation in a ligand-independent manner, ultimately up-regulating genes involved in CRPC tumorigenesis (9).

The primary negative regulator of the PI3K pathway is the tumor suppressor phosphatase and tensin homolog gene (*PTEN*). PTEN is a dual specificity protein and lipid phosphatase that not only targets acidic residues in protein substrates, but more importantly, the 3-phosphate from PIP3, converting it back to PIP2 (10). PTEN signaling regulates cell division and can also direct cells to enter a natural cell death pathway when sufficient growth has taken place by inducing G1-phase cell cycle arrest through the retinoblastoma protein (11, 12). As a regulator of PI3K signaling, loss of *PTEN* leads to over-activation of Akt, which, in turn, is associated with uncontrolled cell proliferation, decreased apoptosis, and enhanced tumor angiogenesis (13). The *PTEN* tumor suppressor gene maps to human chromosome 10q23.3, and this region is known to exhibit high rates of loss of heterozygosity in a variety of human malignancies, including kidney, lung, breast, and prostate cancer (14). In prostate cancer, early reports on the *PTEN* gene focused on small changes of DNA sequence or point mutations that led to inactivation of PTEN protein function (15). In addition, the *PTEN* gene may also be inactivated by epigenetic events such as promoter methylation (16, 17). However in recent years, it has become evident that relatively large deletions and genomic rearrangements affecting *PTEN* are most prevalent in prostate cancer (8, 18). Mechanisms including transcriptional repression, microRNA (miRNA) regulation (19), disruption of competitive endogenous RNA (CeRNA) networks (20), and post translational modifications have also been implicated in the loss of PTEN function and in the initiation of tumorigenesis (21, 22). However, this review will mainly focus on PTEN alterations at the genomic level.

## *PTEN* Deletion Analyses by FISH

Fluorescence *in situ* hybridization (FISH) analyses have provided a robust evaluation of the genomic status of *PTEN* in prostate cancer. This assay design has a centromere probe as a chromosome copy number control, and the *PTEN* locus probe labeled in a different fluorochrome. Using this FISH method, early studies by Yoshimoto et al. (23) analyzing 35 radical prostatectomy specimens showed no *PTEN* deletion in benign glandular epithelium or low-grade Prostatic Intra-epithelial Neoplasia (PIN), while *PTEN* deletions were found in 23% of High-Grade Prostatic Intra-epithelial Neoplasia (HGPIN), a pre-malignant stage of prostate carcinoma, and 68% of overt prostate cancer. The authors concluded that acquisition of a *PTEN* deletion is an important step toward prostatic tumorigenesis (23). Subsequent studies have demonstrated an association between *PTEN* loss and poor clinical outcomes in cohorts ranging from 59 to 322 tumors samples, confirming that *PTEN* alterations confer substantial malignant potential to prostate cancer cells (24–26). Utilizing FISH to determine the hemizygous or homozygous *PTEN* deletion status, Yoshimoto et al. (8) analyzed paired primary adenocarcinomas and regional lymph node metastasis derived from 10 patients and determined that only 1 of the 10 patients retained both copies of the *PTEN* locus in his matched pair biopsy. Hemizygous *PTEN* deletion was found in both the primary and the metastatic nodal tumor samples in 4 of 10 patients, while homozygous *PTEN* deletion was found in both the primary tumor and their metastatic lymph nodes in 3 of the 10 patients. Interestingly, 2 of the 10 patients with a hemizygous *PTEN* deletion in their primary adenocarcinomas, had positive lymph node biopsies that had acquired a homozygous *PTEN* deletion (8). These findings suggest that the transition from one-copy loss to two-copy loss may be associated with metastasis (8). Subsequent supporting studies demonstrated that hemizygous *PTEN* deletions were associated with increased risk and earlier biochemical relapse after radical prostatectomy, whereas homozygous deletions were strongly linked to metastasis and androgen-independent progression (24, 26, 27).

**Figure 2:**
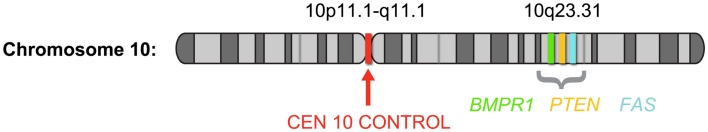
**Schematic depiction of chromosome 10 showing the probe locations of the *PTEN* four-color FISH assay; based on Yoshimoto et al. (29)**. The four-color FISH assay was designed to include *BMPR1A* (green) and *FAS* (aqua) probes flanking either side of the 2.06-Mb minimal deleted region, a *PTEN* probe (orange) at 10q23.3 and a chromosome 10 centromere (red) copy control probe.

Despite being the method of choice for detecting genomic status in FFPE sections, the reported frequency of *PTEN* deletion in prostate cancer tissue using a two-color FISH assay varies widely. To date, reports of *PTEN* deletions ranged from 20 to 60% of localized prostate cancers (24–26). This large range in frequency most likely results from a difference in tissue preparation, stage of disease, and the methodology used to detect the aberration. Several of the studies reported high cutoff values using the two-color technology, and attributed this to artifacts generated during sectioning. During tissue sectioning, part of the cell and nucleus can be sliced out, leading to a “truncation effect” where loss of *PTEN* signal from the nucleus could be scored falsely as gene deletion. It is therefore essential that the false-positives likely to come from truncation be determined by comparison with normal nuclei for all deletion FISH assays (28). One solution for minimizing truncation effects is the use of a four-color FISH assay incorporating additional control loci. Recently, Yoshimoto et al. (29) reported a meta-analysis of 311 published human genome array datasets determined that the minimal prostate cancer-associated *PTEN* deletion at 10q23.3 corresponds to a ∼2.06 Mb region flanked by the *BMPR1A* and *FAS* genes. A four-color FISH assay was designed to include *BMPR1A* and *FAS* probes flanking either side of the minimal deleted region, a *PTEN* probe, and a chromosome 10 centromere copy control probe (29) (Figure 2).

**Figure 3:**
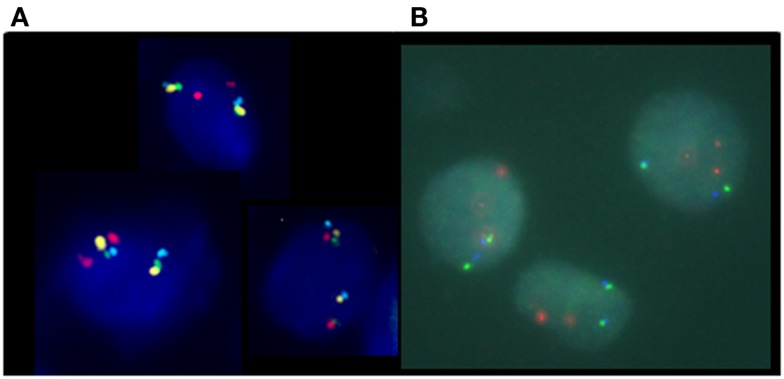
**Phosphatase and tensin homolog gene four-color FISH assay**. **(A)** Normal, non-deleted tissue with red signals showing chromosome 10 centromere regions, green signals showing the presence of BMPR1, blue signal showing the presence of FAS, and yellow signal indicating the presence of the PTEN gene; and **(B)** deleted *PTEN* locus, the yellow signals corresponding to the PTEN gene are absent indicating homozygous deletion of PTEN.

In this four-color FISH assay design, loss of *PTEN* signal, but presence of *BMPR1A* and *FAS* signals, indicates with a higher degree of accuracy that a *PTEN* deletion is present rather than a false-positive resulting from truncation effects. Loss of *PTEN* signal along with loss of flanking probe signals could suggest the presence of an artifact truncation loss. A recent comparison study between a four-color *PTEN* FISH assay and a two-color *PTEN* FISH assay using benign prostatic hyperplasia as a control tissue source for prostate cancer showed that two-color *PTEN* FISH has a mean of 12% of false-positive cells due to truncation losses whereas four-color *PTEN* FISH has a mean false-positive rate of only 4%. Thus, the addition of these control flanking probes provides three-dimensional information in FFPE sections that increases specificity and sensitivity of the assay (29). Representative deleted and normal *PTEN* FISH images are presented (Figure 3).

**Figure 4:**
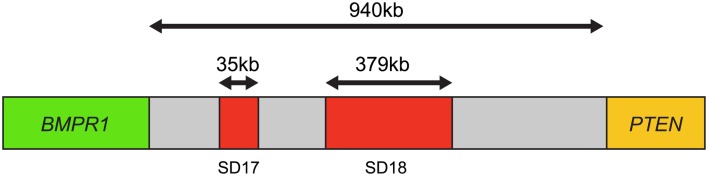
**Map of the breakpoint cluster region between *BMPR1* and *PTEN***. The 940-kb interval between *BMPR1* and *PTEN* includes the segmental duplication clusters SD17 and SD18, which are 35 kb and 379 kb in length, respectively. Both contain clusters of homologous inverted repeat sequences thought to cause genomic instability and mediate PTEN deletion. Based on Yoshimoto et al. (29).

Furthermore, using this four-color FISH assay, breakpoints between *PTEN* and *BMPR1A* or *FAS* were mapped in 100 homozygous and 82 homozygous *PTEN* losses. The results revealed that 69% of the samples had one breakpoint within the 940-kb interval between *BMPR1A* and *PTEN*, suggesting that this interval was a “breakpoint cluster hotspot” (Figure 4).

This region appeared to coincide with segmental duplication sites SD17 and SD18, containing at least 13 homologous inverted repeat sequences over 10 kb in length, which could promote intrachromosomal mis-pairing during homologous DNA repair or DNA replication. This genomic instability is the most likely mechanism by which *PTEN* deletion is mediated in prostate cancer (29).

## PTEN Deletions Detected in Circulating Tumor Cells

Circulating prostate tumor cells are cells that have broken free from the tumor and circulate in the peripheral circulation. Most of these circulating tumor cells (CTCs) that depart from the primary tumor will die, whereas an estimated 0.01% of CTCs are likely to give rise to metastases (30). Despite having important prognostic and therapeutic implications, CTCs have not been widely studied in prostate cancer because of the technical challenges of their detection. Early studies have shown that PSA-positive CTCs from the peripheral blood of patients suffering from multifocal cancers could be subjected to genetic profiling along with tissue isolated from each cancer focus by multiplex PCR-based microsatellite analysis (31–33). Results showed that the mutations detected in CTCs were most likely derived from only one distinct focus, while other foci from the same tumor sometimes had additional LOH. Interestingly, the highest LOH in the PSA-positive CTCs was observed at the *PTEN* locus, suggesting that *PTEN* genetic imbalances might be associated with the occurrence of CTCs in the peripheral blood of patients and with early biochemical recurrence (33). An additional study using FISH analysis of *PTEN* showed heterogeneous loss of *PTEN* in prostate cancer CTCs (34). Their data demonstrated that 6 of 13 patients had homogenous homozygous loss of *PTEN* in all their CTCs, while 7 patients had heterogeneous *PTEN* loss with combinations ranging from a mixture of heterozygous loss/normal *PTEN* to heterozygous/homozygous *PTEN* loss (34). With emerging technology such as the CellSearch detection method, isolation of CTCs from the peripheral blood of patients is now becoming a valid alternative source of tumor tissue that can be subjected to molecular characterization, FISH analyses, and cancer monitoring (35).

## PTEN Deletions and Associated Biomarkers in Prostate Cancer

**Figure 5:**
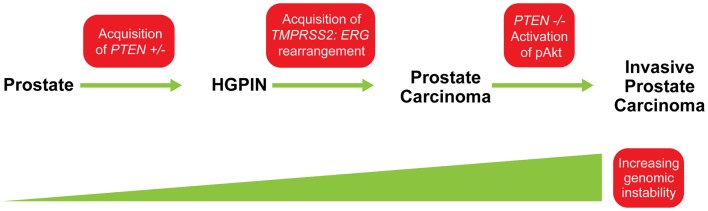
**Model for possible sequence of genomic events in PCA progression**. The acquisition of *PTEN* haplo-insufficiency in prostatic precursor results in a decrease in PTEN protein level, which could lead to genomic instability and the formation of high-grade prostatic intra-epithelial neoplasia (HGPIN). This genomic instability could facilitate ERG rearrangement, and together, synergistic co-operation between PTEN and ERG abnormalities is associated with early steps of PCA formation. Continuing genetic instability generates “PTEN null” subclones and allows the selective advantage for further tumor progression through activation of the Akt pathway. Modified from (24).

The importance of *PTEN* genomic status for prostate cancer prognosis is compelling but understanding its co-operation with other genetic aberrations in the context of this highly heterogeneous disease is crucial for accurately predicting clinical outcomes and developing targeted therapies. One of the most pivotal interactions in both human and murine prostate cancer is that between PTEN and the *ETS*-related gene, *ERG*. Genomic alterations of *ERG* resulting from a fusion with the androgen responsive trans-membrane protease, serine 2 (*TMPRSS2*) gene are highly pervasive in prostate cancer and can be detected in about 40–70% of clinically significant diseases. Several studies have confirmed that tumors with *TMPRSS2:ERG* gene rearrangements are enriched for *PTEN* genomic deletions in localized prostate cancer and CRPC (18, 24, 25, 36). Bismar et al. (37) reported that these two genetic alterations showed significant concurrence within the same tumor focus, ranging between 44 and 71% of hemizygous and homozygous *PTEN* deletions, respectively. King et al. (38) and Carver et al. (39), confirmed this association in a murine model, both documenting that the development of invasive prostate cancer in double-transgenic models occurs at a higher rate than in *ERG* rearrangements or *PTEN* genomic deletions alone. Furthermore, Bismar et al. (24) proposed that the synergistic co-operation between PTEN deletion and ERG rearrangement could be a significant driver for PCA development and tumor progression. A subset of PCA may be driven initially by PTEN genomic instability, which may facilitate the chromosomal rearrangement leading to gene fusion formation and progression of cancer. Subsequent PTEN deregulation by homozygous deletion could then occur to induce invasive PCA possibly through Akt activation (Figure 5). In a recent study using a Tissue MicroArray (TMA), Krohn et al. (40) analyzed *PTEN* status in 2217 hormone naive and 49 hormone-refractory tumors. Their data showed that *PTEN* deletion was strongly associated with *ERG* fusion-positive tumors (29.1 versus 10.7%), suggesting a selective advantage for tumor cells harboring both *PTEN* deletion and *ERG* fusion (40). However, their findings also suggested that *ERG* fusion is not required for *PTEN* loss to determine aggressive tumor behavior, because *PTEN* deletion in both *ERG* fusion-positive and fusion-negative cancers was independently linked to poor prognosis, while the presence of *ERG* fusion was unrelated to patient prognosis (40). Collectively, these FISH studies of *PTEN* gene loss and *ERG* gene rearrangements could be pursued for patient stratification and patient management.

The AR is a steroid receptor member of the larger nuclear receptor superfamily, and plays a central role in normal prostate development as well as in prostate cancer initiation and progression. Androgen deprivation is currently the standard therapy for metastatic prostate cancer, but patients invariably relapse with a more aggressive CRPC. It has been widely recognized that AR signaling remains important even in the presence of reduced androgen levels and thus remains a major target for targeted therapeutic interventions. In 43 primary prostate cancer samples, Choucair et al. (41) determined that AR expression was significantly lower in prostate cancer tumors harboring a *PTEN* deletion compared to those with no *PTEN* deletion. However, the underlying mechanism associating decreased AR expression in the presence of *PTEN* deletion is still unclear (41). El Sheikh et al. (42) also showed in 47 samples of hormone naive prostate cancers that reduced expression of either biomarker was associated with the development of androgen independence and reduced survival in patients whose tumors remained androgen dependent (42). In contrast, Sircar et al. (26) reported a positive correlation between *PTEN* deletion status and up-regulation of *AR* expression in CRPC patients. These results suggest that *AR* expression levels differ according to the stage of the disease, with genomic amplification of AR likely to occur in CRPC, but rarely in untreated prostate cancer (43, 44). Therefore, *AR* gene amplification may play a direct role in prostate cancer progression and may be associated with failure of androgen deprivation therapy (ADT). Since *AR* expression levels are low in hormone naive tumors, addition of an inhibitor of PI3K/mTOR to the standard ADT of advanced prostate cancer may therefore be beneficial to patients with *PTEN* deleted tumor. A number of other studies have implicated differential expression of *AR* and *PTEN* deletions/Akt activation as significant predictors for disease progression and the development of CRPC (37, 44, 45). Their data suggest that assessment of *PTEN*/*AR* co-expression proves useful in distinguishing prostate cancer with a more favorable prognosis from those with a high likelihood of developing recurrent disease.

The proto-oncogene *MYC* has been associated with cell transformation. It is known that overexpression of MYC can immortalize human prostatic epithelial cells, so gain of function of MYC is clearly an oncogenic factor in human prostate cancer. Comparative Genomic Hybridization (CGH) studies have showed that gain of 8q, including 8q24 involving the *MYC* gene, is one of the most frequent alterations in prostate cancer (46). In a comprehensive study using FISH with probes for *MYC* (8q24), ∼40% of primary tumors and >90% of metastases showed varying levels of *MYC* copy number gains (47). Whereas primary tumors typically have simple gain of *MYC* due to an extra copy of 8q, metastases have more frequent regional *MYC* amplification, suggesting that *MYC* is more commonly involved in prostate cancer progression (15). Recently, Zafarana et al. (48) reported for the first time the combined role of *PTEN* loss and *MYC* gain using global high-resolution CGH analyses validated by FISH in a cohort of 126 intermediate-risk prostate cancer patients after receiving radiotherapy. They reported that copy number alterations in *PTEN* (allelic loss) and *MYC* (allelic gain) were associated with significantly increased genetic instability and biochemical relapse compared to tumors with normal *PTEN* and *MYC* status. In light of these results, triaging patients by the use of *PTEN* and *MYC* copy number changes within pre-treatment biopsies may allow for better use of systemic therapies to target sub-clinical metastases or locally recurrent disease and improve clinical outcomes (48).

## *PTEN* Mutation and Methylation Analysis

In addition to allelic deletion, functional loss of PTEN can also be caused by mutations and epigenetic modifications. Some of the most frequent mutations identified in *PTEN* are frameshift or non-sense mutations that lead to inactivation of the protein (Table 1). In localized prostate cancer, the incidence of *PTEN* mutations has been found to occur at <20%, a frequency notably lower than that of *PTEN* deletions (∼40% of localized cancers) (2, 49–53). However, in advanced and metastatic disease, *PTEN* appears to be more frequently mutated in up to 60% of patients who had multiple metastases as well as in cell lines and xenografts derived from metastatic prostate cancer (7, 54). *PTEN* appears to be more frequently mutated in metastases of prostate cancer, providing further evidence for the idea that PTEN plays a crucial role in the progression of prostate cancer (55). Recent advances in massively parallel DNA sequencing technologies, allowing for the identification of base substitutions as well as indels and copy number alterations at greater sensitivity and cost effectiveness than screening by traditional Sanger sequencing, have great promises to offer a new strategy for personalized cancer care. Several studies have shown that targeted mutational and copy number analyses in advance prostate cancer using Next-Generation Sequencing (NGS) were able to detect PTEN somatic point mutations as well as genomic deletion in up to 44% of analyzed patient samples (2, 56, 57). The comprehensive genomic profiling that NGS technologies offer is an exciting new tool that will not only help in the discovery of new biomarkers but also in the design of personalized therapies in the context of such a heterogeneous disease. Epigenetic inactivation of the *PTEN* promoter has been identified in multiple types of cancers such as thyroid, lung, cervical, and breast cancer (58). Even though several genes such as *APC*, *GSTP1*, and *MGMT*, are known to be highly methylated in prostate cancer (59–61), to date, limited data has shown the occurrence of *PTEN* methylation in prostate cancer. One study using prostate cancer xenografts, demonstrated by Northern analysis and RT-PCR that the level of *PTEN* mRNA expression was reduced or absent in five of the nine xenografts studied. The mRNA expression was then restored with the demethylating agent 5-azadeoxycytidine in at least one of the five xenografts suggesting that methylation of CpG islands in the *PTEN* genomic locus was responsible for decreased *PTEN* expression. However, it is also possible that the effect of 5-azadeoxycytidine on *PTEN* is mediated indirectly through a second gene, such as a transcription factor that may be the true target of methylation. Direct evidence that the *PTEN* gene is methylated in prostate cancer is still required (17, 62).

**Table 1:** **Mutations in the *PTEN* gene identified in prostate cancer**.

Exon	Position	Predicted effect	Reference
Exon 1	G20STOP	Non-sense	Dong et al. (55)
Exon 2	R55G	Missense	Dong et al. (55)
Exon 3	T38G	Inactivation	Krohn et al. (40)
Exon 5	E91Q	Inactivation	Suzuki et al. (7)
	R387STOP	Non-sense	Suzuki et al. (7)
	H118Y	Inactivation	Krohn et al. (40)
	I101A	Missense	Dong et al. (55)
	I135V	Missense	Dong et al. (55)
	Q150G	Missense	Dong et al. (55)
	Q110STOP	Non-sense	Dong et al. (55)
	P95S	Missense	de Muga et al. (63)
	A164STOP	Non-sense	de Muga et al. (63)
Exon 6	564	Non-sense	Cairns et al. (49)
Exon 7	c.761–765del	Frameshift	Cairns et al. (49)
	c.672–673Ins	Non-sense	Suzuki et al. (7)
	c.224Ins	Frameshift	Suzuki et al. (7)
	D223N	Missense	de Muga et al. (63)
Exon 8	E201STOP	Non-sense	Krohn et al. (40)
	D326N	Inactivation	Krohn et al. (40)
	H272Y	Missense	Dong et al. (55)
Exon 9	T348I	Missense	Dong et al. (55)
	K344R	Missense	Dong et al. (55)
	T382S	Missense	Dong et al. (55)

## Emerging Anticancer Therapies for PTEN-Deficient Prostate Cancers

Because PTEN is a tumor suppressor that negatively regulates the PI3K pathway, up-regulation of this pathway’s downstream targets is commonly observed in PTEN-deficient prostate cancers. Both Akt and mTOR are two important PI3K targets that are frequently activated in human primary prostate cancer specimens, as evidenced by increased phosphorylation of both Akt and S6RP, a downstream target of mTOR (64, 65). The development of specific inhibitors for either one of these kinases has thus emerged as important cancer therapeutic targets. It was reported that inhibition of mTOR by rapamycin analogs such as ridaforolimus or temsirolimus showed antitumor effects in clinical studies (65–68). MK-2206 has also recently emerged as a potent allosteric inhibitor of Akt and is currently in Phase 1 trial studies for the treatment of prostate cancer (69). Recently, Zhang et al. (70) showed that inhibition of either mTOR with ridaforolimus or Akt with MK-2206 alone had no effect on the status of the other kinase in castrate-sensitive settings. However, simultaneous inhibition of both mTOR and Akt demonstrated additive antitumor effects (70). These findings strongly indicate that the mTOR signaling network in the PTEN-null tumor is independent of Akt activity and that both Akt and mTOR downstream signaling pathways play a part in PTEN-deficient prostate tumors. These results strongly support the rationale for using Akt and mTOR inhibitors in combination therapy (ClinicalTrials.gov identifier NCT01295632).

Most recently, the mTOR inhibitor drug Everolimus was evaluated in a Phase 2 trial as a first-line treatment in patients with mCRPC (ClinicalTrials.gov identifier NCT00976755) (71). Everolimus has been reported to inhibit tumor growth in human prostate cancer cell lines as well as in transgenic mice expressing human Akt (72). Everolimus treatment was also shown to increase sensitivity to mTOR inhibition in tumors cells with PTEN loss *in vitro* (73, 74). In this study 35% of chemotherapy-naïve patients with mCRPC treated with a daily dose of 10 mg of single-agent Everolimus showed progression-free survival (PFS) at the primary end point of 12 weeks. Thirty percent of analyzable tumor samples were found to have a *PTEN* deletion determined using FISH, and those in particular showed a trend toward longer PFS after treatment. Despite modest activity in some patients with mCRPC, Everolimus is a promising candidate drug for treating PTEN-deleted prostate tumors, and further investigations of this drug in combination with other therapies are warranted.

Targeting AR through androgen ablation therapy is the mainstay of prostate cancer treatment. However, these cancers often progress and, as a result, treatment options become limited. While often termed “androgen-independent,” recent work has shown that AR signaling remains critical throughout the course of the disease (75). This hypothesis has been validated by the clinical successes of a new generation of potent AR antagonists such as MDV3100 or antiandrogen agents such as bicalutamide and abiraterone (76, 77). Recently, it has also been demonstrated that the AR and PI3K signaling pathways are feedback regulated in a reciprocal manner (78). That is, androgen independence, commonly associated with *PTEN* loss, is associated with the activation of PI3K signaling; conversely, PI3K inhibitors can reactivate androgen signaling and sensitivity to antiandrogen therapy. In particular, recent studies have identified several mechanisms for the crosstalk between AR by mTOR (79–81). In light of these findings, Squillace et al. (82) recently demonstrated that dual inhibition of AR and mTOR with bicalutamide and ridaforolimus, respectively, had synergistic antiproliferative effect in prostate cancer cells *in vitro* when compared with each agent alone (82).

The combination therapy resulted in full inhibition of each pathway and exhibited potent antitumor activity with parallel reductions in plasma PSA levels in xenograft models. Taken together, ridaforolimus and bicalutamide represent a potentially effective combination strategy for PTEN-deficient prostate cancer therapy. Considering the success of these new compounds and their mechanisms of action, the identification of *PTEN* deletions has the potential to be a useful companion diagnostic assay for therapeutics targeting the PIK3/mTOR pathway.

## Conclusion

Over the past decade, extensive research has led to a more detailed understanding of the molecular mechanism(s) governing the initiation and progression of prostate cancer. Although significant progress has been made in our ability to forecast outcomes for prostate cancer after therapy using clinical and histological variables, the ability to accurately predict response to a specific treatment remains elusive. Molecular and cytogenetic assays such as FISH analyses of *PTEN* have paved the way to a much clearer understanding of cancer status and disease progression. With the improved design of the four-color FISH assay, *PTEN* genomic status can be used as a reliable diagnostic tool and potential companion diagnostic for emerging anticancer drugs. Overall it is clear that the status of PTEN is a powerful biomarker that promise effective diagnosis and improved patient stratification and management.

## Conflict of Interest Statement

The authors declare that the research was conducted in the absence of any commercial or financial relationships that could be construed as a potential conflict of interest.
